# Supplementary figures and images for: Immunoglobulin profile and B‐cell frequencies are altered with changes in the cellular microenvironment independent of the stimulation conditions
Source: Immun Inflamm Dis. 2020 Jul 8;8(3):458–67. doi: 10.1002/iid3.328 (PMC7416019; doi:10.1002/iid3.328)

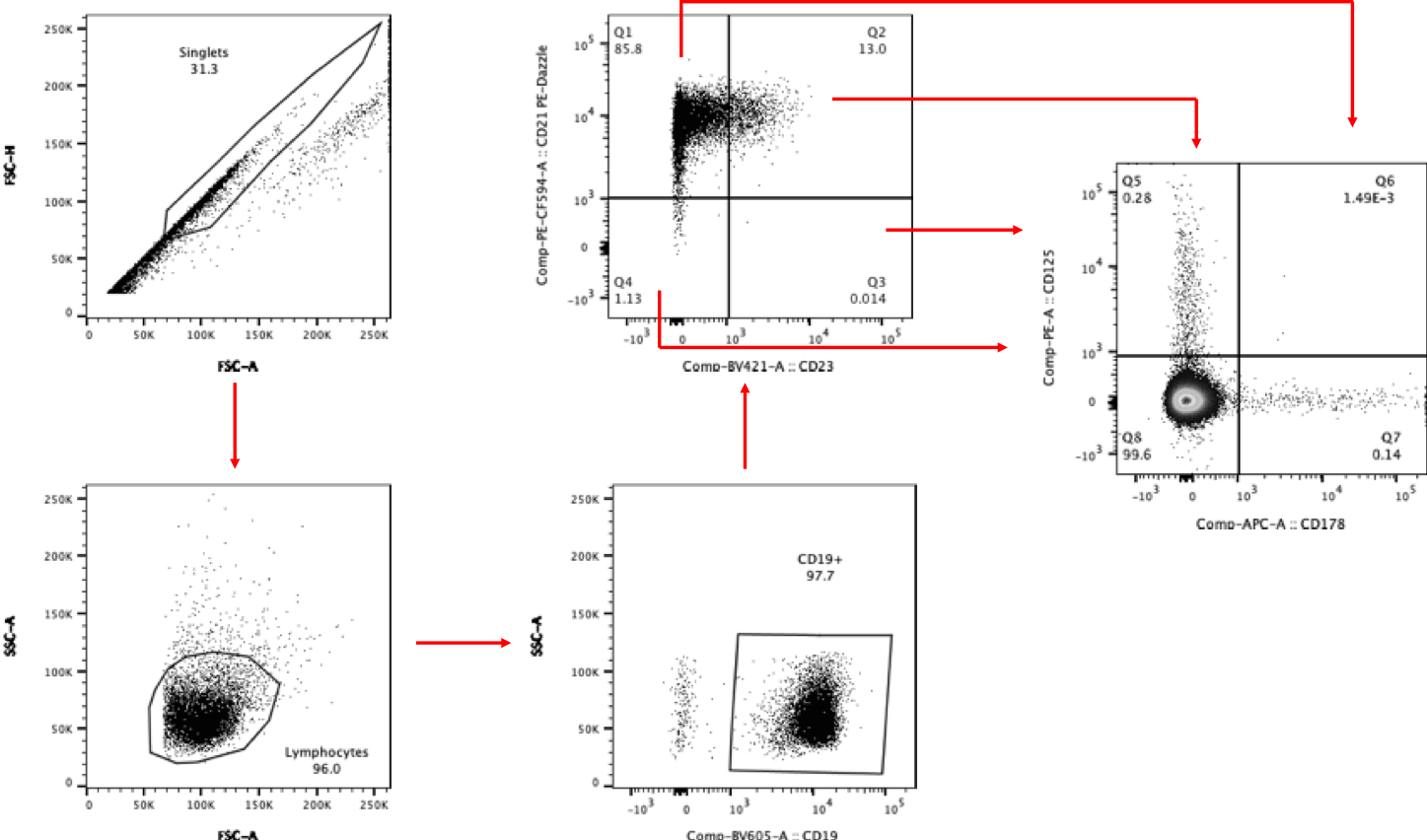

Supplement: Supplementary file 1 — Supplementary Figure 1. Gating strategy for T1, T2, MZ and FO B‐cells [file IID3-8-458-s001.png]
